# The two sides of Tat

**DOI:** 10.7554/eLife.12686

**Published:** 2016-01-19

**Authors:** Matjaz Barboric, Koh Fujinaga

**Affiliations:** 1 Department of Biochemistry and Developmental Biology, Faculty of Medicine, University of Helsinki, Helsinki, Finlandmatjaz.barboric@helsinki.fi; 2 Department of Medicine, University of California, San Francisco, San Francisco, United Stateskoh.fujinaga@ucsf.edu

**Keywords:** transcription factors, chromatin, epigenetics, RNA polymerase II, transcription, Human

## Abstract

A virus protein called Tat plays a dual role in HIV infection by regulating the expression of genes belonging to the virus and genes belonging to the host cells.

**Related research article** Reeder JE, Kwak Y-T, McNamara RP, Forst CV, D’Orso I. 2015. HIV Tat controls RNA Polymerase II and the epigenetic landscape to transcriptionally reprogram target immune cells. *eLife* **4**:e08955. doi: 10.7554/eLife.08955

**Image** HIV (yellow) attacking a human immune cell (blue) (Image: S Pincus, E Fischer, A Athman; NIAID/NIH)

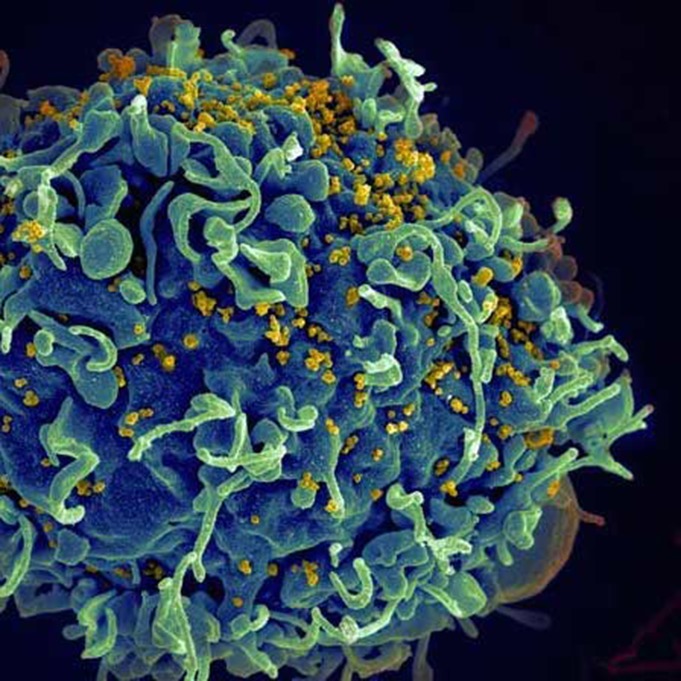

Like all viruses, human immunodeficiency virus (HIV) must engage with the cells of its host to be able to replicate. Compared to other retroviruses, this interaction is particularly complex in the case of HIV because its genome encodes many proteins that take advantage of various cell processes and counteract host defenses. One of these proteins is called transactivator of transcription (Tat) and it is essential for the expression of HIV genes in host cells. Now, in eLife, Ivan D’Orso of the University of Texas Southwestern Medical Center (UTSW) and colleagues – including Jonathan Reeder and Youn-Tae Kwak of UTSW as joint first authors – demonstrate that Tat can also regulate the expression of many host genes (Reeder et al., 2015).

In the first stage of gene expression, a gene is “transcribed” to make messenger RNA (mRNA) by an enzyme called RNA polymerase II (Pol II). After Pol II binds to the promoter region of a gene and starts to make an mRNA molecule, the enzyme stops due to the actions of pause-inducing factors. The ability of Pol II to continue to transcribe the gene (in a step called elongation) depends on a cellular protein complex called P-TEFb (short for positive transcription elongation factor b). In the case of HIV, the situation is very similar. Pol II pauses soon after initiating from the promoter of the viral genome producing only short mRNAs. However, Tat can override this transcriptional block by recruiting P-TEFb to Pol II via a RNA stem-loop structure called the transactivation response element, which is found at the beginning of all virus mRNAs (Ott et al., 2011).

While most researchers have focused on understanding how Tat activates the transcription of HIV, some studies have demonstrated that Tat also alters the expression of host genes to promote the production and spread of HIV. For example, Tat manipulates immune T cells in its host to allow the virus to infect and replicate in them (Li et al., 1997, Huang et al., 1998). Moreover, Tat induces the expression of chemokines that attract uninfected T cells and macrophages, facilitating expansion of HIV in the host (Izmailova et al., 2003). On the other side, Tat can also contribute to the depletion of T cells during AIDS progression by up-regulating cellular pro-apoptotic genes (Kim et al., 2010). Finally, Tat is able to repress the transcription of several genes encoding receptors of the innate immune system responses. However, we still do not have a comprehensive view of the host genes that Tat targets, or how Tat alters the transcription of these genes.

Reeder, Kwak, D'Orso and two colleagues – Ryan McNamara (UTSW) and Christian Forst (Icahn School of Medicine at Mount Sinai) – took on this challenge by analyzing T cells that produced the Tat protein with a series of genome-wide approaches. They identified close to 3000 sites in the human genome that are occupied by Tat. The presence of Tat changed the expression of about 2000 host genes, and almost quarter of these were classified as direct targets because Tat bound to their promoters or to other sites in the genes. About half of these direct targets were up-regulated by Tat, while the rest were down-regulated. Further experiments revealed that for a subset of the target genes, Tat is able to regulate either the initiation or elongation by Pol II.

Next, Reeder, Kwak et al. investigated how Tat can modulate different steps in the transcription of host genes. For example, to stimulate or inhibit the start of transcription, Tat regulates the loading Pol II to cellular promoters. Of note, at the majority of up-regulated genes, Tat appears to facilitate Pol II promoter presence from sites within gene bodies by inducing DNA looping. However, to regulate elongation, Tat can either promote or prevent the recruitment of P-TEFb to specific genes. Importantly, Reeder, Kwak et al.’s further findings suggest that, unlike what happens with viral genes, Tat does not seem to rely on RNA structures similar to the HIV transactivation response element to regulate target host genes. Instead, Tat co-opts some of the master transcriptional regulators of its host that also control cellular gene transcription in uninfected cells.

In agreement with an earlier report (Marban et al., 2011), Reeder, Kwak et al. found that binding sites for the master regulator called ETS1 were, among others, most frequent near the genomic regions that Tat occupies. Tat interacts with ETS1 protein and knocking down the *ETS1* gene abolished the recruitment of Tat to some target genes, thus preventing it from being able to modulate their transcription. Moreover, ETS1 motifs are found at both up-regulated and down-regulated Tat targets, underscoring that gene-specific circumstances determine the effect of Tat. Curiously, HIV itself contains an ETS1 binding site upstream of its promoter, and this allows ETS1 to stimulate viral gene expression in collaboration with other transcription factors of the cell (Sieweke et al., 1998; Yang et al., 2009).

The report by Reeder, Kwak et al. arguably represents the most thorough characterization of the role of Tat on host gene expression. While making sure that the HIV genome is transcribed, Tat also fine-tunes two critical steps in the expression of specific cellular genes. Is this tweaking of host gene transcription a necessary survival strategy for HIV, or merely a side effect of the presence of potent transcriptional regulator Tat in the cell nucleus? Or has this possible side effect been transformed through evolution into a winning strategy of the virus? Whatever the correct answer might be, Reeder, Kwak et al. confirmed that the expression of many Tat target genes changed as expected during HIV infection. Moreover, many of these genes appear to be involved in signaling pathways and biological processes that could help HIV to spread in the host.

This study raises further questions. Does Tat modulate the expression of hundreds of previously identified host proteins that interact with HIV gene products or are critical to replication of the virus? Is Tat also capable of regulating the termination of transcription at host genes? How exactly do the interactions between Tat, master transcriptional regulators like ETS1, and host proteins lead to changes in gene expression? Further, has HIV devised one strategy to promote very high levels of transcription of its own genome, while exploiting cellular transcriptional regulators to provoke only modest (but beneficial) changes in the expression of host genes? Answers to these and other questions shall advance our understanding of how and why HIV Tat reprograms its host.

## References

[bib1] Hollenhorst PC, Chandler KJ, Poulsen RL, Johnson WE, Speck NA, Graves BJ, Snyder M (2009). DNA specificity determinants associate with distinct transcription factor functions. PLoS Genetics.

[bib2] Huang L, Bosch I, Hofmann W, Sodroski J, Pardee AB (1998). Tat protein induces human immunodeficiency virus type 1 (hIV-1) coreceptors and promotes infection with both macrophage-tropic and t-lymphotropic HIV-1 strains. Journal of Virology.

[bib3] Izmailova E, Bertley FMN, Huang Q, Makori N, Miller CJ, Young RA, Aldovini A (2003). HIV-1 tat reprograms immature dendritic cells to express chemoattractants for activated t cells and macrophages. Nature Medicine.

[bib4] Kim N, Kukkonen S, Gupta S, Aldovini A, Rice AP (2010). Association of tat with promoters of PTEN and PP2A subunits is key to transcriptional activation of apoptotic pathways in HIV-infected CD4+ t cells. PLoS Pathogens.

[bib5] Li CJ, Ueda Y, Shi B, Borodyansky L, Huang L, Li Y-Z, Pardee AB (1997). Tat protein induces self-perpetuating permissivity for productive HIV-1 infection. *Proceedings of the National Academy of Sciences USA*.

[bib6] Marban C, Su T, Ferrari R, Li B, Vatakis D, Pellegrini M, Zack JA, Rohr O, Kurdistani SK, Rossi JJ (2011). Genome-wide binding map of the HIV-1 tat protein to the human genome. PLoS ONE.

[bib7] Ott M, Geyer M, Zhou Q (2011). The control of HIV transcription: keeping RNA polymerase II on track. Cell Host & Microbe.

[bib8] Reeder JE, Kwak Y-T, McNamara RP, Forst CV, D'Orso I (2015). HIV tat controls RNA polymerase II and the epigenetic landscape to transcriptionally reprogram target immune cells. eLife.

[bib9] Sieweke MH (1998). Cooperative interaction of ets-1 with USF-1 required for HIV-1 enhancer activity in t cells. The EMBO Journal.

[bib10] Yang H-C, Shen L, Siliciano RF, Pomerantz JL (2009). Isolation of a cellular factor that can reactivate latent HIV-1 without t cell activation. Proceedings of the National Academy of Sciences USA.

